# The balance of adult mental health care: provision of core health versus other types of care in eight European countries

**DOI:** 10.1017/S2045796018000574

**Published:** 2018-10-17

**Authors:** G. Cetrano, L. Salvador-Carulla, F. Tedeschi, L. Rabbi, M. R. Gutiérrez-Colosía, J. L. Gonzalez-Caballero, A.-L. Park, D. McDaid, R. Sfetcu, J. Kalseth, B. Kalseth, Ø. Hope, M. Brunn, K. Chevreul, C. Straßmayr, G. Hagmair, K. Wahlbeck, F. Amaddeo, for the REFINEMENT group

**Affiliations:** 1 Social Care Workforce Research Unit, King's Policy Institute, King's College London, London, UK; 2 Department of Neurosciences, Biomedicine and Movement Sciences, University of Verona, Verona, Italy; 3 Centre for Mental Health Research, Research School of Population Health, College of Health and Medicine, Australian National University, Canberra, Australia; 4 PSICOST Research Association, Jerez de la Frontera, Spain; 5 Departamento de Psicología, Universidad Loyola Andalucía, Sevilla, Spain; 6 Department of Statistics and Operations Research, University of Cadiz, Cádiz, Spain; 7 Personal Social Services Research Unit, Department of Health Policy, London School of Economics and Political Science, London, UK; 8 Institute for Economic Forecasting, Bucharest, Romania; 9 Faculty of Psychology and Educational Sciences, University Spiru Haret, Bucharest, Romania; 10 Department of Health Research, SINTEF, Trondheim, Norway; 11 Université Paris Diderot, Sorbonne, Paris, France; 12 Inserm, ECEVE, U1123, F-75 010, Paris, France; 13 AP-HP, URC-Eco, Paris, France; 14 IMEHPS.research, Vienna, Austria; 15 Department for Cultural Analysis, Universitaet Klagenfurt, Klagenfurt, Austria; 16 Department of Mental Health, National Institute for Health and Welfare (THL), Helsinki, Finland

**Keywords:** Community mental health, health service research, mental health, psychiatric services

## Abstract

**Aims:**

Although many mental health care systems provide care interventions that are not related to direct health care, little is known about the interfaces between the latter and core health care. ‘Core health care’ refers to services whose explicit aim is direct clinical treatment which is usually provided by health professionals, i.e., physicians, nurses, psychologists. ‘Other care’ is typically provided by other staff and includes accommodation, training, promotion of independence, employment support and social skills. In such a definition, ‘other care’ does not necessarily mean being funded or governed differently. The aims of the study were: (1) using a standard classification system (Description and Evaluation of Services and Directories in Europe for Long Term Care, DESDE-LTC) to identify ‘core health’ and ‘other care’ services provided to adults with mental health problems; and (2) to investigate the balance of care by analysing the types and characteristics of core health and other care services.

**Methods:**

The study was conducted in eight selected local areas in eight European countries with different mental health systems. All publicly funded mental health services, regardless of the funding agency, for people over 18 years old were identified and coded. The availability, capacity and the workforce of the local mental health services were described using their functional main activity or ‘Main Types of Care’ (MTC) as the standard for international comparison, following the DESDE-LTC system.

**Results:**

In these European study areas, 822 MTCs were identified as providing core health care and 448 provided other types of care. Even though one-third of mental health services in the selected study areas provided interventions that were coded as ‘other care’, significant variation was found in the typology and characteristics of these services across the eight study areas.

**Conclusions:**

The functional distinction between core health and other care overcomes the traditional division between ‘health’ and ‘social’ sectors based on governance and funding. The overall balance between core health and other care services varied significantly across the European sites. Mental health systems cannot be understood or planned without taking into account the availability and capacity of all services specifically available for this target population, including those outside the health sector.

## Introduction

For several decades, mental health policies and practices across Europe have focused on shifting the balance from hospital-based to integrated community-based services (Thornicroft and Tansella, 2003, 2004; Knapp *et al*., 2011). In most developed countries, the balance and mix of services have changed in response to new policy directions and to demands for more individualised psycho-social interventions (Thornicroft and Tansella, 2005). For example, personalisation is a prominent policy aspiration in England, aiming to enhance choice and control for people using both health and social care services (Larsen *et al*., 2013). Moreover, there is a growing interest in recovery-oriented treatments within mental health. Thus, the relevance of interventions to promote social re-engagement, such as getting a job, or making new friends, or learning new skills, has been acknowledged (Slade, 2009; Knapp *et al*., 2014; Slade *et al*., 2017). All these initiatives were further enhanced by approval of the World Health Organization (WHO) ‘Framework on Integrated People-Centred Health Services’ in 2016. This highlighted the need to rebalance care provision towards inclusive community care by promoting care integration and coordination across provider settings (WHO, 2016). The emergence of the discipline of ‘health care delivery science’ advocating for better standards and tools for the international comparison of universal access and service variation across geographical areas (Mulley *et al*., 2013) has also been helpful.

Despite this reorientation, discussion of the funding, planning and delivery of European mental health services is not grounded in evidence from actual comparisons of integrated service availability and capacity across geographical areas. It is therefore important in any comparative analysis of mental health across Europe to incorporate service provision delivered outside the health care sector and understand the interfaces between these other services and core health care (McDaid *et al*., 2007).

The boundaries between service provision delivered through the health system and those delivered elsewhere are far from clear and can vary substantially within and across countries. What is regarded as a health *v*. a non-health or social service also depends on country-specific regulations and financing mechanisms (Straßmayr *et al*., 2013). There are also different levels of decentralisation and devolution for health and other services. These differences may mean that if the focus is on health care systems, two countries with similar mixes of services may appear to have very different levels of service availability, depending on the extent to which services lie within the health care system (McDaid *et al*., 2007). Problems of comparability are even more evident when exploring interfaces with services provided in other sectors.

Moves towards a standardised classification system to facilitate descriptions of mental health services across different geographical areas and settings have evolved over the last two decades. For example, the Description and Evaluation of Services and Directories in Europe for Long Term Care (DESDE-LTC), an evolution of the European Service Mapping Schedule (ESMS) for the evaluation of services in mental health (Johnson *et al*., 2000), was developed as a means of standardising descriptions and classifications of services (Salvador-Carulla *et al*., 2006, 2013).

DESDE-LTC is used to distinguish between core health and other care provision. ‘Core health care’ refers to services whose explicit aim is direct clinical treatment (in this case for mental health problems) usually provided by health professionals with over 3 years of training in health sciences (i.e., physicians, nurses, psychologists, physiotherapists) (WHO, 2006). ‘Other care’ is typically provided by other staff and its main aim is not direct and highly specialised clinical treatment. It typically includes accommodation, training, promotion of independence and autonomy, case management, employment support and social skills. It also implies more integration, inclusion, social participation and encouragement of mental, as well as social capital, within communities (Slade *et al*., 2014; Thornicroft *et al*., 2011, 2016). This classification draws on similar constructs used elsewhere, such as by the International Classification of Functioning, Disability and Health (ICF) (WHO, 2013), which distinguishes between ‘health professionals’ and ‘other professionals’. The International Standard Classification of Occupations (ISCO-08) (ILO, 2012) identifies different health workforce subgroups (mainly health and associate professionals) according to assumed differences in skill level and specialisation required to fulfil job tasks and duties. This approach has also been followed by the System of Health Accounts (SHA 2.0) produced by OECD, Eurostat and WHO (OECD, 2011).

The definition ‘other care’ (or ‘associated care’ according to the OECD terminology) does not necessarily mean being funded or governed differently. The DESDE-LTC typology concentrates on the main activity provided by the service rather than funding source and governance structure. Such a functional approach is innovative as it avoids traditional comparisons based on the agency/department responsible for the oversight and governance of specific services. These have proved problematic due to wide geographical variations across European mental health services. Therefore, the DESDE-LTC classification allows for separate analysis and comparison of groups of services undertaking similar activities and provided in similar settings to facilitate territorial comparisons of like-with-like (Salvador-Carulla *et al*., 2006, 2013; Gutierrez-Colosía *et al*., 2017).

Undertaken as part of the European Union funded REFINEMENT project (REsearch on FINancing systems’ Effect on the quality of MENTal health care) (http://www.refinementproject.eu), which compared differences in financing mechanisms to understand their impact on the quality and efficiency of European mental health systems, the two main aims of this specific analysis were:

1. To use DESDE-LTC to identify and compare core health and other care services provided to people with mental health problems in selected areas of eight European countries.
2. To investigate the balance of care by analysing the types (residential, outpatient, day care), target group (mental health *v*. general health) and characteristics (staff and bed availability) of services that provide core health and other care in selected areas.

## Method

### Instruments

This present paper draws on data collected within the REFINEMENT project using the REMAST tool (Refinement Mapping Services Tool). This tool has five main sections (Table 1), with the focus on the MHSI (Mental Health Services Inventory) used to classify and describe mental health services in the eight study areas. The core of the MHSI is represented by DESDE-LTC, which provides most of the information needed to complete this inventory. The feasibility, reliability and validity of DESDE-LTC has previously been described (Salvador-Carulla *et al*., 2000, 2011*a*, 2011*b*, 2013; Gutierrez-Colosía *et al*., 2017).

**Table 1.** Study instruments and data collection procedure

REMAST (REFINEMENT Mapping Services Tool)	(a) Population Data; (b) Verona Socio-economic Status (SES) Index; (c) Mental Health System Checklist describing policies and organisation of mental health care; (d) Mental Health Services Inventory (MHSI) using the DESDE-LTC instrument; (e) Geographical Data (Salvador-Carulla *et al*., 2015).
BSIC (Basic Stable Inputs of Care)	BSICs are the minimal service organisation units that can be identified in the organisation of care, usually composed of an administrative unit with an organised set of structures and professionals.
MTC (Main Type of Care)	MTC is the major descriptor of the BSIC in relation to its more relevant activity. The DESDE-LTC includes 90 MTCs or codes for the classification of BSICs.
Example of BSIC and MTC	A rehabilitation service within a general hospital that has a specific team of professionals, independent budget and management is classified as a BSIC. One BSIC may be described by more than one MTC, for example, a rehabilitation service offering long-term residential care with 24 h physician cover outside the hospital as well as outpatient care on a biweekly basis is described by MTCs R7 and O9.1.
Data collection procedure	Specific training was provided on the use of the DESDE-LTC classification system to the researchers of the REFINEMENT project responsible for data gathering and codification. For the collection of local information in every study area, researchers consulted available databases and health agencies and established direct contact with services. Particularly, in Girona (Spain) and Helsinki and Uusimaa (Finland), there were formal agreements with the Departments of Health. Data were gathered into an *ad hoc* database, which was completed in August 2012, and further revised and updated to create a final database in July 2013. Detailed information on the procedure is provided in Salvador-Carulla *et al*. (2015).

### Study areas

Each of the eight countries (Austria, England, Finland, France, Italy, Norway, Romania and Spain) selected a study area with a population between 200 000 and 1 500 000 inhabitants, preferably not limited to a macro-urban area within a municipality. Detailed characteristics of the study areas are available on the REFINEMENT webpage (http://www.refinementproject.eu) and in Gutierrez-Colosía *et al*. (2017).

### Units of analysis

We focused on universally accessible publicly funded services that provided mental health care to adults (+18) meeting ICD-10 (International Classification of Diseases) F20–F69 disease classification criteria (WHO, 2010). This excluded services only accessible with fully private insurance or out-of-pocket payment without public reimbursement. Services for people with an ICD-10 diagnosis of organic mental disorders (F00-F09), psychoactive substance use (F10-19), intellectual disability (F70-79) and those for child and adolescent disorders were excluded as they are provided by a separate system in many countries. To classify and compare services, the operational definitions of Basic Stable Inputs of Care (BSIC) and Main Type of Care (MTC) were used (Table 1).

### Data analysis

All MTCs in the study areas were analysed separately according to whether they provided core health or other care. Table 2 illustrates DESDE-LTC codes included in each of the two groups.

**Table 2.** Taxonomy of core health and other care provision for adults with mental health problems

Health care	Residential	Acute, 24 h physician cover	R0, R1, R2	*Units from general hospitals, psychiatric hospitals, other specialist hospitals*
		Acute, non-24 h physician cover	R3.0, R3.1.1	*Some acute wards at specialised psychiatric hospitals without 24* *h medical cover*
		Non-acute, 24 h physician cover	R4, R5, R6, R7	*Units for rehabilitation, community therapeutic programmes, nursing homes*
	Outpatient	Acute, health-related care^a^	O1.1, O2.1, O3.1, O4.1	*Emergency units in general hospitals, home and mobile teams which provide crisis treatment*
		Non-acute, health-related care^a^	O5.1, O6.1, O7.1, O8.1, O9.1, O10.1	*Community mental health teams, outpatient psychiatric clinics, single-handed psychiatrists and psychologists*
	Day care	Acute	D1.1, D1.2, D10	*Day hospitals*
		Non-acute, non-work structured, health-related care	D4.1, D8.1	*Day care centres*
Other care	Residential	Non-acute, non-24 h physician cover	R8, R9, R10, R11, R12, R13, R14	*Residences, houses for groups, therapeutic communities with various levels of support from staff*
	Outpatient care	Acute, not meeting the criteria for health-related care	O1.2, O2.2, O3.2, O4.2	*Crisis teams in the community for homeless people*
		Non-acute, not meeting the criteria for health-related care	O5.2, O6.2, O7.2, O8.2, O9.2, O10.2	*Home care for daily activities (e.g., cleaning, grooming, cooking, toileting and dressing)*
	Day care	Non-acute, work	D2, D6	*Sheltered work services or opportunities on the open labour market*
		Non-acute, work-related care	D3, D7	*Occupational centres, workshops*
		Non-acute, non-work structured, education, social, cultural or other non-work structured care	D4.2, D4.3, D4.4, D8.2, D8.3, D8.4	*Creative activities, art, music, group work*
		Non-acute, non-structured care	D5, D9	*Social contact, practical advice and/or support*

a Main goal is the specific clinical care and at least 20% of the staff is qualified health care professionals.

Both at the overall level and for each study area, the total number of MTCs providing health and other care and the number of MTCs per type of care and target group were computed, as well as the percentage of core health care and other care services as a share of total MTCs. In each study area, the rate of MTCs classified as core health care and other care per 100 000 adult population was also calculated. Services were considered ‘general health’ if they could be used by other users alongside mental health service users, whereas services with ‘mental health’ as target group were those providing care exclusively to people with mental health problems. General practitioners were not included in this analysis.

As for staff/bed availability, only data about services exclusively targeted at mental health service users were considered, since those for general health users were affected by missing data and our focus was on mental health provision.

The overall percentage of staff in core health and other MTCs was calculated, alongside the full-time equivalent staff rate per 100 000 adult population in each area. The percentages of each professional category in total staff numbers were determined for health and other MTCs. A composite indicator for multidisciplinary staff was created. Following Burns's (2004) definition, where there was at least one physician, one nurse, one psychologist and one social worker or occupational therapist, this was classified as multidisciplinary. Self-employed specialists were not included in this indicator since they typically work single-handedly. The global percentage of beds in all areas in health and other care MTCs was computed. Finally, in each area, the average, minimum and maximum number of beds per residential unit was considered, along with the total number of beds per 100 000 adults.

## Results

### Core health *v.* other care provision

**Fig. 1.**
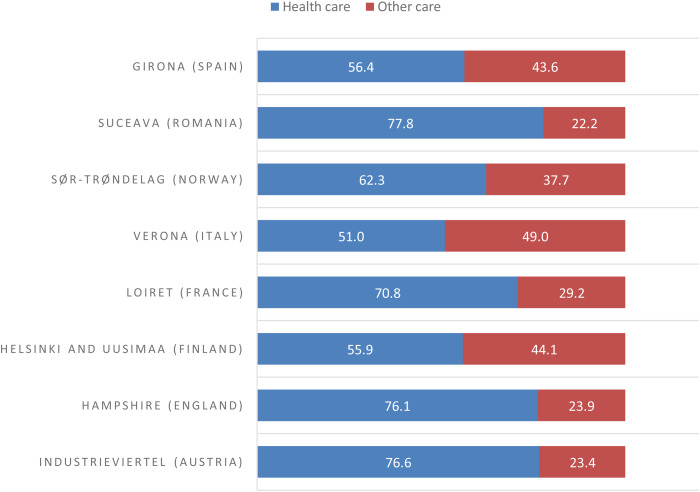
Balance of care for people with mental health problems in eight European study areas.

Table 3 presents the distribution of health and other care services in the study areas. The total number of MTCs in the eight study areas was 1270. Overall, 822 MTCs (65%) provided core health care and 448 (35%) other care. Such ratios ranged from 22% in Suceava to 49% in Verona (Fig. 1).

**Table 3.** Comparison of core health and other care services in the field of mental health in eight European study areas

	Number of MTCs^a^	MTCs per 100 000 adult inhabitants^a^	Type of care (*N* of MTCs)			Target group (*N* of MTCs)	
			Residential	Outpatient^a^	Day care	Mental health	General health
**Industrieviertel (Austria)**							
Health care	128 (108)	28.8 (24.3)	8	118 (108)	2	122	6
Other care	39	8.8	17	8	14	34	5
**Hampshire (England)**							
Health care	83	6.1	25	57	1	83	
Other care	26	1.9	4	6	16	25	1
**Helsinki and Uusimaa (Finland)**							
Health care	143	11.9	66	58	19	143	
Other care	113	9.4	85	5	23	113	
**Loiret (France)**							
Health care	187 (113)	43.8 (26.4)	9	158 (113)	20	187	
Other care	77	18.0	49	11	17	15	62
**Verona (Italy)**								
Health care	75	19.1	17	36	22	49	26
Other care	72	18.3	29	41	2	22	50
**Sør-Trøndelag (Norway)**							
Health care	147 (34)	65.3 (15.1)	15	131 (34)	1	114	33
Other care	89	39.5	2	38	49	35	54
**Suceava (Romania)**							
Health care	28 (18)	4.9 (3.2)	5	21 (18)	2	28	
Other care	8	1.4	8			8	
**Girona (Spain)**								
Health care	31	5.2	11	16	4	16	15
Other care	24	4.0	11		13	24	
**All study areas**							
Health care	822 (273)	15.7^b^	156	595 (273)	71	742	80
Other care	448	8.6^b^	205	109	134	276	172
*Total*	*1270*	*24.3*^b^	*361*	*704*	*205*	*1018*	*252*

a The numbers in brackets refer to single-handed psychiatrists and psychologists.

b Such figures mainly reflect the results in the larger study areas.

In total, 156 residential services (MTCs) were classified as providing core health care and 205 other care. Looking at outpatient services, significantly more services were identified as providing health (595 MTCs) than other care (109 MTCs). This included 273 MTCs for single-handed psychiatrists and psychologists mapped in Industrieviertel, Loiret, Sør-Trøndelag and Suceava. Furthermore, 71 day care services (MTCs) were reported as health and 134 as other care related. Looking at the target group, 1018 were MTCs targeted specifically at people with mental health problems, while 252 were for generic users where at least 20% of users had a mental health problem. In most areas, day and residential care seemed to be the main forms of other care provision. The rate of ‘core health care’ and ‘other care’ services per 100 000 adult population varied from 4.9 and 1.4 in Suceava to 65.3 and 39.5 in Sør-Trøndelag, respectively.

In both Verona and Sør-Trøndelag, outpatient services made up a substantive proportion of other care services for people with mental health problems. This component was delivered by municipalities in Norway and local authority social services in Italy. Although such municipality services also operate in the other study areas, it was not possible to map them due to a lack of information.

### MTCs characteristics

Table 4 shows the distribution of staff in health and other care services in the eight study areas. Overall 79% of staff worked in health care services and 21% in other care.

**Table 4.** Staff in core health and other care services (MTCs) in eight European study areas^a^

	Total staff (FTE) per 100 000 inhabitants in the study area	Number of physicians (% of total staff)	Number of psychologists (% of total staff)	Number of nurses (% of total staff)	Number of social workers (% of total staff)	Number of occupational therapists (% of total staff)	Number of other staff (% of total staff)	% of services with multidisciplinary staff^b^
**Industrieviertel (Austria)**								
Health care	63.8	36.4	16.5	33.4	7.3	2.9	3.4	28.6
Other care (2)^c^	22.3	0.1	14.8	32.9	4.4	3.3	44.5	0.0
**Hampshire (England)**								
Health care (13)^c^	128.1	9.0	7.6	61.9	8.1	6.7	6.7	88.6
Other care (20)^c^	2.6	5.7	0.0	0.0	2.9	2.9	88.6	0.0
**Helsinki and Uusimaa (Finland)**								
Health care	184.4	12.9	6.2	48.0	5.1	3.6	24.2	60.1
Other care (19)^c^	63.5	0.6	0.3	14.9	1.3	0.7	82.2	0.0
**Loiret (France)**								
Health care (1)^c^	154.1	12.0	14.2	46.6	2.7	0.04	24.5	39.7
Other care	6.4	2.2	1.0	77.3	1.9	0.0	17.6	20.0
**Verona (Italy)**								
Health care	118.3	15.7	4.4	37.5	3.2	0.0	39.3	30.6
Other care	37.6	6.6	5.6	6.4	2.4	0.0	79.0	22.7
**Sør-Trøndelag (Norway)**								
Health care (6)^c^	359.8	9.9	19.3	40.7	5.6	4.5	20.1	25.7
Other care (5)^c^	73.0	0.1	1.0	18.1	27.3	2.3	51.2	0.0
**Suceava (Romania)**								
Health care	36.4	22.9	13.5	53.0	5.3	4.8	0.5	90.0
Other care	78.6	5.4	2.0	26.7	1.8	17.0	47.2	62.5
**Girona (Spain)**								
Health care (1)^c^	29.4	31.8	7.4	22.2	9.7	1.7	27.3	46.7
Other care (6)^c^	6.7	0.9	10.5	0.4	9.8	2.5	76.0	27.8

a Only services targeted exclusively at people with mental health problems were included here (*N*  =  1018).

b Staff including at least one physician, one nurse, one psychologist and one social worker or occupational therapist. Single-handed professionals are not included in this indicator.

c The number in brackets refers to services for which data on staff are missing.

Beginning with health-related services, Sør-Trøndelag had the highest number of total staff per 100 000 adult population (359.8). In other care, this rate ranged from 2.6 in Hampshire to 78.6 in Suceava. Some rates may be underestimated due to missing data, especially in Hampshire where about 30% of services had insufficient data on staff.

In general, the staff rate was higher in health care rather than other services, with the exception of Suceava where the opposite was found.

In most areas, healthcare staff were predominantly nurses, including Hampshire, Helsinki and Uusimaa, Loiret, Sør-Trøndelag and Suceava. In Industrieviertel and Girona, physicians dominated, while in Verona, other staff were most common followed by nurses. In services that were not directly health-related, other staff comprised the highest number of staff in all areas, apart from Loiret where nurses predominated.

Multidisciplinary teams seemed to be a frequent model of health care provision in all areas. Suceava showed the highest percentage of services with multidisciplinary staff, both for health (90.0%) and other care (62.5%). After Suceava, Hampshire showed the highest proportion of health services with multidisciplinary staff (88.6%). Multidisciplinary teams were also common in health services in the Finnish area, with 60.1% of services having this element in place. In the other areas, this percentage ranged from 25.7 to 46.7%. A multidisciplinary pattern was less common in services not providing core health care and missing in Industrieviertel, Hampshire, Helsinki and Uusimaa, Sør-Trøndelag. However, missing data mean figures need cautious interpretation.

Table 5 shows great diversity among areas in availability of residential beds for people with mental health problems. In total, 45% of beds were located in health care services and 55% in services not providing direct health care. As far as health care services are concerned, the average number of beds per unit ranged from 13.9 in Verona to 75.0 in Suceava. In other care services, this average ranged from 3.4 in Loiret to 106.4 in Suceava. The highest rates of health care beds per 100 000 adult inhabitants were found in Helsinki and Uusimaa and Sør-Trøndelag. In other care services, the highest rates of beds were found in Helsinki and Uusimaa and Suceava.

**Table 5.** Provision of core health and other care in residential MTCs in eight European study areas^a^

	Average number of beds per residential service (Min–Max)	Total beds per 100 000 adult inhabitants in the study area
**Industrieviertel (Austria)**		
Health care	52.0 (44–60)	23.4
Other care	13.8 (2–46)	37.1
**Hampshire (England)**		
Health care	18.4 (4–75)	33.7
Other care	25.5 (11–43)	7.5
**Helsinki and Uusimaa (Finland)**		
Health care	16.7 (3–56)	91.3
Other care	24.4 (6–91)	172.2
**Loiret (France)**		
Health care	29.8 (17–77)	62.7
Other care	3.4 (1–5)	11.2
**Verona (Italy)**		
Health care	13.9 (10–25)	42.5
Other care	6.4 (1–20)	35.8
**Sør-Trøndelag (Norway)**		
Health care	15.9 (5–41)	105.7
Other care	10.0 (10–10)	8.9
**Suceava (Romania)**		
Health care	75.0 (40–160)	65.9
Other care	106.4 (10–420)	149.5
**Girona (Spain)**		
Health care	44.7 (42–50)	22.4
Other care	11.8 (4–58)	21.7

a Only services targeted exclusively at people with mental health problems were included here.

## Discussion

To our knowledge, this paper presents the first comprehensive analysis of mental health care provision in Europe following a holistic approach and using a standardised methodology based on service function or activity and not on name, funding or governance systems. In line with previous reports (WHO, 2011, 2014), our results show great diversity in provision of mental health services across Europe. As McDaid *et al*. (2007) point out, our findings demonstrate that a substantial part of mental health resources in Europe are delivered in services not classified as ‘core health care’. Our data also indicate that other care services play different roles in different countries, resulting in heterogeneous configurations of mental health care provision across Europe. Such diversity was further reflected in great variation among study areas in the availability of services per capita.

Looking at residential bed availability in health and other care services, Industrieviertel, Hampshire, Verona and Girona reported similar and relatively low rates, with Girona having the lowest bed rate in health care services. This pattern may be representative of a typology of care with a stronger community approach (Gutierrez-Colosía *et al*., 2017). In Hampshire, community mental health teams were the predominant way of providing health care services, with multidisciplinary staff being highly prevalent in most services. Moreover, in this area, day care services, mainly run by non-governmental organisations (NGOs) to support social functioning, seemed to play a significant role in providing non-direct health-related care.

Verona showed the highest percentage of services identified as not providing core health care, with nearly half of all mapped services being found in other care settings, including non-acute residential services with various levels of support, home care teams and day services in the community (Amaddeo *et al*., 2012).

Different patterns were found in Helsinki and Uusimaa, Loiret, Sør-Trøndelag and Suceava. Helsinki and Uusimaa reported the second highest bed rate in health services and highest in other care; residential services also represented the major area of other care provision here. The majority of beds were found in nursing homes with 24 h staffing providing permanent care for people with severe mental health problems. The remainder were mainly beds in nursing homes with less intensive daily support. These categories of beds have been rapidly increasing in Helsinki and Uusimaa and represent trans-institutionalisation (a shift from hospitals to other institutions), as well as private entrepreneurship (the majority of nursing homes are private for-profit companies under public contract and highly profitable) (Pedersen and Kolstad, 2009). Such findings had important practical implications in Finland and led to changes in resource allocation (Gutierrez-Colosía *et al*., 2017).

The rate of beds in health care units in Loiret was quite high compared with other countries, and highest if only acute beds in hospital settings were considered. This is representative of the French system where hospital care still plays a significant role (Verdoux, 2007). Interestingly, when looking at other care services in Loiret, not many services were targeted at people with mental health problems, as the majority of services were generic and could be used by anyone with no further specification. Finally, the high prevalence of nursing staff in other care services seems indicative of a rather health-orientated mental health system.

Sør-Trøndelag reported the highest bed rate in residential units providing health care, represented by both acute and non-acute hospital-based services. Half of the beds were in new mental health facilities providing specialist health care with a more community-oriented focus (District Psychiatric Centres) (Gutierrez-Colosía *et al*., 2017). This Norwegian area also had the highest staff rate in health care services. These two figures are indicative of a system with a high availability of community, residential and hospital services.

Suceava had a high number of beds both in health and other care services, mostly indefinite stay beds with daily support. This area also had the highest percentage of services with multidisciplinary staff. However, one possible explanation for these results is that these services consist mainly of large institutions where many categories of staff work. Also, in Romania there is no catchment area organisation and mental health services usually serve the whole country population (Junjan *et al*., 2009). The data adjusted for the study area population must therefore be interpreted as an approximation rather than a clear indicator of service use and availability. Future studies are needed to analyse balance of care patterns, including service availability and capacity in Eastern European countries, with a particular focus on the deinstitutionalisation process and mental health care reforms.

Typologies of services specific to certain areas were also found. For example, in Industrieviertel, Loiret, Sør-Trøndelag and Suceava, single-handed psychiatrists and psychologists were an important organisational component of the mental health system. In Sør-Trøndelag and Verona, municipalities and local authority social services played a substantial role in providing care to people with mental health problems.

We also identified some ‘grey zones’ that were more difficult to map and analyse. We are referring here to day care/rehabilitation activities and supported housing. For example, in Norway supported housing took the form of apartments rented by service users who then received mobile support from staff working for municipalities. Even though the apartments typically were co-located with personnel services that provide 24 h access to care, these were considered home-based services and not institutional/residential care and hence were coded as outpatient care and not as community residential care as in other areas where residential homes were managed by public agencies or NGOs.

### Strengths and limitations

Our study involved the use of an internationally standardised instrument for service assessment, online training materials, a brief face-to-face training course and monitoring of data collection by the coordinating group. This study provides multi-country, multi-site analysis of patterns and balance of mental health care provision across Europe. This is a major advance over previous studies that compared services availability between areas in two countries such as Spain and Italy (Salvador-Carulla *et al*., 2005), Spain and Finland (Sadeniemi *et al*., 2018), Spain and Chile (Salvador-Carulla *et al*., 2008), and Norway and Russia (Dahl *et al*., 2017). This methodology can be used in future studies for longitudinal monitoring of service change within an area, for monitoring mental health reform in different world regions, as well as being applied to other areas of integrated care, such as for older people. However, the large number of researchers needed to collect data across the eight partner countries, variable levels of information available in different databases in these countries, as well as the practicality of the assessment tool and the complexity of assessment of mental health systems and integrated care may have led to data inconsistencies. The identification of the minimal units of care at every service required considerable time, effort and revision of the information gathered in each study area. A further limitation relates to missing information, particularly in ‘other care’ services. For example, a consistent proportion of services in the English study area had missing information on staff. Moreover, the differences found in services availability and characteristics among the study areas do not necessarily represent differences at the country level.

Furthermore, population-based figures should be interpreted with caution as in some countries, such as Austria and Romania, there are no health care catchment areas. Especially in densely populated areas with short travel distances, patients from outside the geographical study area may use a service in the study area, and, *vice versa*, patients from the selected study area may use services outside the area.

Another limitation lies in the terminology itself. The term ‘other care’ is broad and services have been reported here to a varying degree. The definition of ‘service’ and on how beds are reported may differ between countries. Differences may arise both related to how services are organised and funded and how they are reported in national statistics (Kalseth *et al*., 2013). In order to overcome these terminological problems, we have produced an international glossary of terms for health systems research in health care (Montagni *et al*., 2017). Furthermore, data on availability and care capacity should be completed with information on financing, demand for and outcomes of services. These aspects have been analysed in other work packages of the REFINEMENT project (Kalseth *et al*., 2013).

Finally, a fully bottom-up approach was missing in our study and not all areas surveyed included full mapping of all available services. In some instances, with hindsight, the only way to obtain information on the individual non-health care services would have been by contacting mental health advocacy groups and/or service user groups. Hence, for future studies, a triangulation of data, involving relevant stakeholder groups including service users and carers, is recommended.

## Conclusion

Better coordination and integration of health and other care services are urgently needed in Europe (Valentijn *et al*., 2013). However, very little information is available on the overall availability and capacity of mental health provision. Developing harmonised mental health data across Europe is an essential step towards better mental health care (Wahlbeck, 2011). This paper presents the first cross-national comparison of services for mental health care focusing on the balance between ‘core health care’ and ‘other care’. The distinction between health and other care provided in our study overcomes the traditional division between ‘health’ and ‘social’ services which is mainly based on service governance. Our paper offers an original taxonomy built on the DESDE-LTC instrument which enables classification of services according to core activities provided.
